# HHV Predicting Correlations for Torrefied Biomass Using Proximate and Ultimate Analyses

**DOI:** 10.3390/bioengineering4010007

**Published:** 2017-01-24

**Authors:** Daya Ram Nhuchhen, Muhammad T. Afzal

**Affiliations:** Mechanical Engineering Department, University of New Brunswick, Fredericton, NB E3B 5A3, Canada; mafzal@unb.ca

**Keywords:** biomass, torrefaction, higher heating value, proximate analysis, ultimate analysis, correlations

## Abstract

Many correlations are available in the literature to predict the higher heating value (HHV) of raw biomass using the proximate and ultimate analyses. Studies on biomass torrefaction are growing tremendously, which suggest that the fuel characteristics, such as HHV, proximate analysis and ultimate analysis, have changed significantly after torrefaction. Such changes may cause high estimation errors if the existing HHV correlations were to be used in predicting the HHV of torrefied biomass. No study has been carried out so far to verify this. Therefore, this study seeks answers to the question: “Can the existing correlations be used to determine the HHV of the torrefied biomass”? To answer this, the existing HHV predicting correlations were tested using torrefied biomass data points. Estimation errors were found to be significantly high for the existing HHV correlations, and thus, they are not suitable for predicting the HHV of the torrefied biomass. New correlations were then developed using data points of torrefied biomass. The ranges of reported data for HHV, volatile matter (VM), fixed carbon (FC), ash (ASH), carbon (C), hydrogen (H) and oxygen (O) contents were 14.90 MJ/kg–33.30 MJ/kg, 13.30%–88.57%, 11.25%–82.74%, 0.08%–47.62%, 35.08%–86.28%, 0.53%–7.46% and 4.31%–44.70%, respectively. Correlations with the minimum mean absolute errors and having all components of proximate and ultimate analyses were selected for future use. The selected new correlations have a good accuracy of prediction when they are validated using another set of data (26 samples). Thus, these new and more accurate correlations can be useful in modeling different thermochemical processes, including combustion, pyrolysis and gasification processes of torrefied biomass.

## 1. Introduction

Biomass is widely-available renewable energy resource with balanced CO_2_ emissions and absorption. However, for the proper use of biomass resources, their physical, chemical and thermodynamic properties play an essential role in designing energy systems [1]. For instance, the higher heating value (HHV), which gives the energy content of biomass, is considered to be an important fuel parameter for designing a combustion system [2]. The HHV refers to the total energy released by a kg of fuel when it is completely burnt out. The experimental procedure, as it requires a properly insulated adiabatic bomb calorimeter, for determining the HHV of a fuel is burdensome [3]. Therefore, having an accurate correlation is always an asset for a design engineer. There are many correlations to predict the HHV of raw biomass using the proximate and ultimate analyses. A detailed review on such correlations has been presented in Moreno et al. [3]. However, the use of such correlations would only be appropriate if the estimation errors were in the acceptable range. Estimation of errors between the model predicted and the measured HHV of biomass is expressed using different parameters, such as the mean absolute error (MAE), the average absolute error (AAE) and the average bias error (ABE) [1,2,4,5,6,7,8,9,10,11,12].

To devise HHV predicting empirical correlations using multiple variables’ linear or non-linear regression analysis of many data points, one can consider HHV as a dependent parameter and the components of proximate (volatile matter, fixed carbon and ash contents) and ultimate analyses (carbon, hydrogen and oxygen contents) as independent parameters. Akkaya [10] has adopted this approach, which assumes HHV as a function of two, three and four independent variables, to develop proximate analysis-based empirical correlations. Among all of the analyzed HHV correlations, Akkaya [10] concluded that the correlation with four independent variables (moisture, fixed carbon, volatile matter and ash contents) has the least error of estimation and can be used for the future. Parikh et al. [7] have also initially proposed the various form of correlations that include both linear and non-linear effects of different components of the proximate analysis. They then selected empirical correlation with all three components of the proximate analysis because it has the minimum prediction error. A similar methodology was also adopted by Nhuchhen and Salam [1] to derive HHV predicting correlation using all components of the proximate analysis in the ratio forms.

However, one may argue that the selection of correlations with all components of proximate and ultimate analyses may not be the correct approach, as the fixed carbon content or the oxygen content may be replaced by the linear combination of other components. As it will not improve the prediction power of correlation, such correlations with all components of proximate and ultimate analyses can be avoided. Another aspect of developing empirical correlations is the use of a wide range of data points. As the basis of the derivation of HHV predicting correlations is only a statistical analysis of the data points incorporated in the analysis, such correlations will not be valid beyond the range of the used data points. In addition, the correlation derived for a limited type of species or materials will not be applicable for other types of materials. This study, thus, excludes the correlations with all of the components of proximate and ultimate analyses and uses only the data points collected for the torrefied biomass produced from various biomass species.

Recently, research on biomass torrefaction that can enhance the biomass fuel characteristics has increased significantly. Many studies have shown that the torrefaction process, a thermal pretreatment method, increases the HHV, hydrophobicity, grindability and combustion properties. Studies have found that the torrefaction of biomass has changed components of both the proximate and ultimate analyses. Typically, the dry torrefaction process is carried out at atmospheric pressure condition in a temperature range of 200–300 °C and in an inert environment, whereas the wet torrefaction process deploys a reactor with highly pressurized water in relatively low-temperature conditions (180–230 °C) [13]. The changes in the properties of torrefied biomass depend on different operating and design parameters, such as temperature, pressure, residence time, working media, particle size, type of feedstock and reactor types. For instance, a high torrefaction temperature leads to more devolatilization reactions and causes a more solid mass loss compared to that at low temperature. More on the effect of torrefaction on biomass and the technologies of torrefaction are reviewed in different publications [13,14,15,16,17].

While torrefaction reduces the percentage of volatile matter, it increases the percentage of the fixed carbon and ash contents in the torrefied biomass. This increases the fuel ratio (fixed carbon to volatile matter contents) of biomass and decreases the char reactivity [18]. This could lead to a more stable combustion process of the torrefied biomass compared to that of the raw biomass. In the same manner, the torrefaction process reduces the oxygen to carbon (O/C) and hydrogen to carbon (H/C) ratios of biomass and makes biomass more compatible with coal.

Given the major changes in the proximate and ultimate analyses of the torrefied biomass, it may be erroneous to use the existing HHV correlations, which were developed using raw biomass, for predicting the HHV of the torrefied biomass. Though one may argue that the existing expressions, which are valid for a wide range of biomass materials, may also be useful to determine the HHV of the torrefied biomass, this study thus examines and confirms if the existing correlations based on the proximate and ultimate analyses can be used or not. At the time of writing this paper, no such correlations, which use HHV, proximate analysis and ultimate analysis of torrefied biomass, are also reported in the literature. This study, therefore, includes (i) reviewing the published literature on biomass torrefaction and collects the information on proximate and ultimate analyses, (ii) reviewing the published correlations to predict the HHV of raw biomass, (iii) examining if the currently available correlations can be used to predict HHV of the torrefied biomass or not, (iv) developing new forms of correlations for predicting HHV using a large number of published data points of the proximate and ultimate analyses for the torrefied biomass and (v) validating the selected correlations with another set of data.

## 2. Materials and Methods

Different published papers were reviewed to gather the information on proximate, ultimate and heating value analyses of both the raw and torrefied biomass materials. The collected information (in dry basis) from the literature for torrefied and raw biomass materials are summarized in Table S1 [12,19,20,21,22,23,24,25,26,27,28,29,30,31,32,33,34,35,36,37,38,39,40,41,42,43,44,45,46,47] and Table S2 [2,7,9,12,19,20,21,22,23,24,25,26,27,28,30,31,32,33,34,35,37,38,39,40,41,42,43,44,45,46,47,48,49,50,51,52,53,54,55,56,57,58,59,60], respectively. Both tables are provided in the supplementary file. Before validating the existing HHV correlations, HHV values of raw and torrefied biomass were plotted with different components of the proximate and ultimate analyses to get a visual insight.

In order to validate if the existing correlations can be deployed or not, only a few selected existing HHV correlations were tested to predict the HHV of the torrefied biomass from Table S1. To ensure all types of existing correlations get tested, different types of correlations that contain (a) only one component; (b) only two components; (c) three or more components and (d) non-linear terms of proximate and ultimate analyses were selected for testing purposes. Estimation errors were calculated for the selected existing correlations using data from Table S1. Disagreements between the predicted and the measured HHV of torrefied biomass were analyzed by calculating the estimation errors. More discussions are presented in Section 3.2.

There could be a number of possible new forms of correlations that can predict the HHV of torrefied biomass. Thus, the authors have used various new forms of correlations to incorporate the individual and combination effects of different components of the proximate and ultimate analyses. Table 1 presents all of the new form of correlations analyzed in this study. Constant terms a, b, c, d, e, f, g and h are determined using the principle of the least sum square error between the measured and predicted HHV values of torrefied biomass materials. Constant terms were initially guessed and then iterated to minimize the sum of square errors ($\overset{N}{\underset{i=1}{\sum }}{\left(P_{i}-M_{i}\right)}^{2}$).

### Estimation Errors

The correlation is said to be the best-fitted regression line if the error of the estimation tends to zero [1]. However, it would be not possible to have such correlations. Therefore, three forms of estimation errors, including the mean absolute error (MAE), average absolute error (AAE) and average biased error (ABE), were calculated to select statistically-appropriate HHV correlations. All of the estimation errors are determined as:

$$\mathrm{MAE}=\overset{N}{\underset{i=1}{\sum }}\left|P_{i}-M_{i}\right|/N$$

$$\mathrm{AAE}=\left(\overset{N}{\underset{i=1}{\sum }}\left|P_{i}-M_{i}\right|/M_{i}\right)/N$$

$$\mathrm{ABE}=\left(\overset{N}{\underset{i=1}{\sum }}\left(P_{i}-M_{i}\right)/M_{i}\right)/N$$

where P and M represent the predicted and measured HHV of the biomass sample, respectively. N (246) is the number of sample data used for the regression analysis. While the AAE measures the degree of closeness between the predicted and measured HHV values, the ABE tells the degree of overestimation and underestimation of the HHV values. On the other hand, the MAE provides the amount of error in the same unit that the physical quantity has. This study has considered the correlation with the lowest MAE value as a probable best correlation. Therefore, the predicted HHV values will not be exactly the same as the experimentally-measured data. Different studies [1,2,4,5,6,7,8,9,10,11,12] have adopted this approach of analyzing the estimation errors for developing empirical correlations to predict the HHV of biomass and coals.

## 3. Results and Discussion

### 3.1. Scatter Distribution of Data

**Figure 1:**
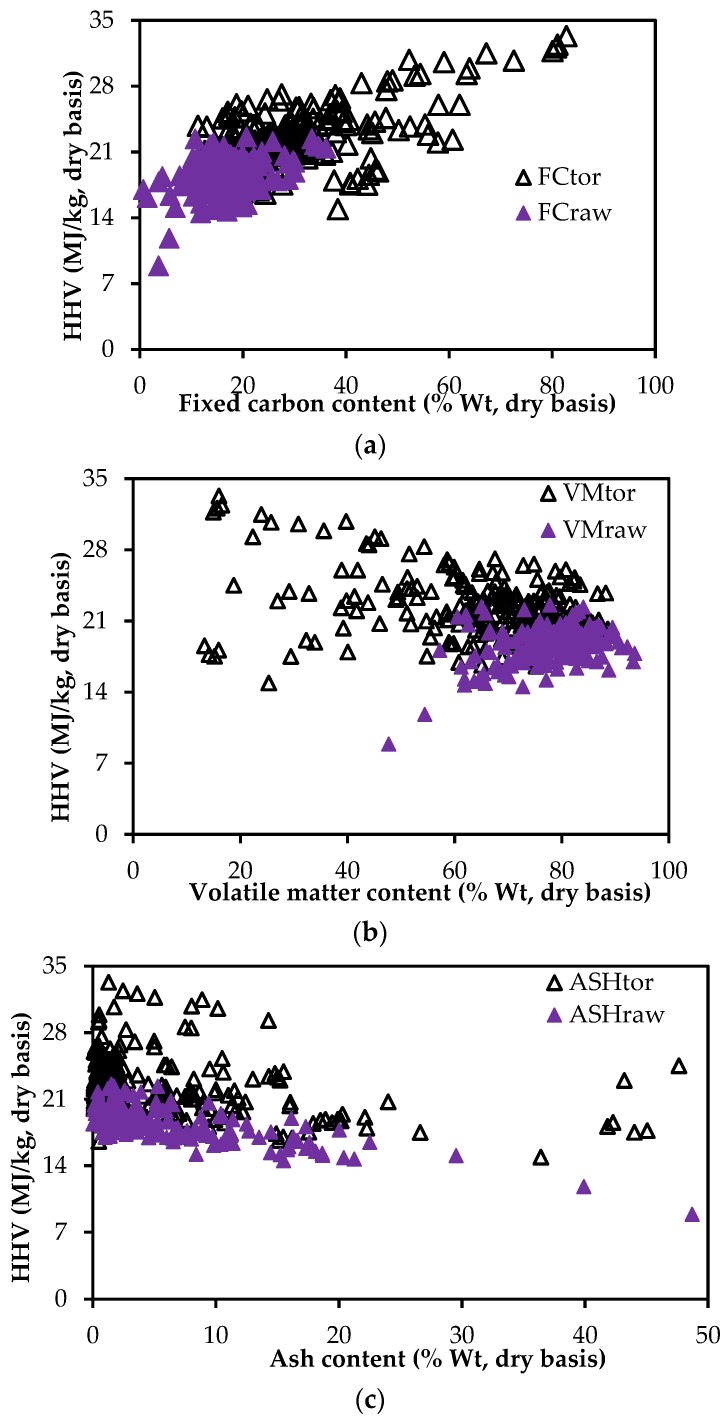
Variation of the HHV values of raw and torrefied biomass: (**a**) fixed carbon content; (**b**) volatile matter content; and (**c**) ash content.

**Figure 2:**
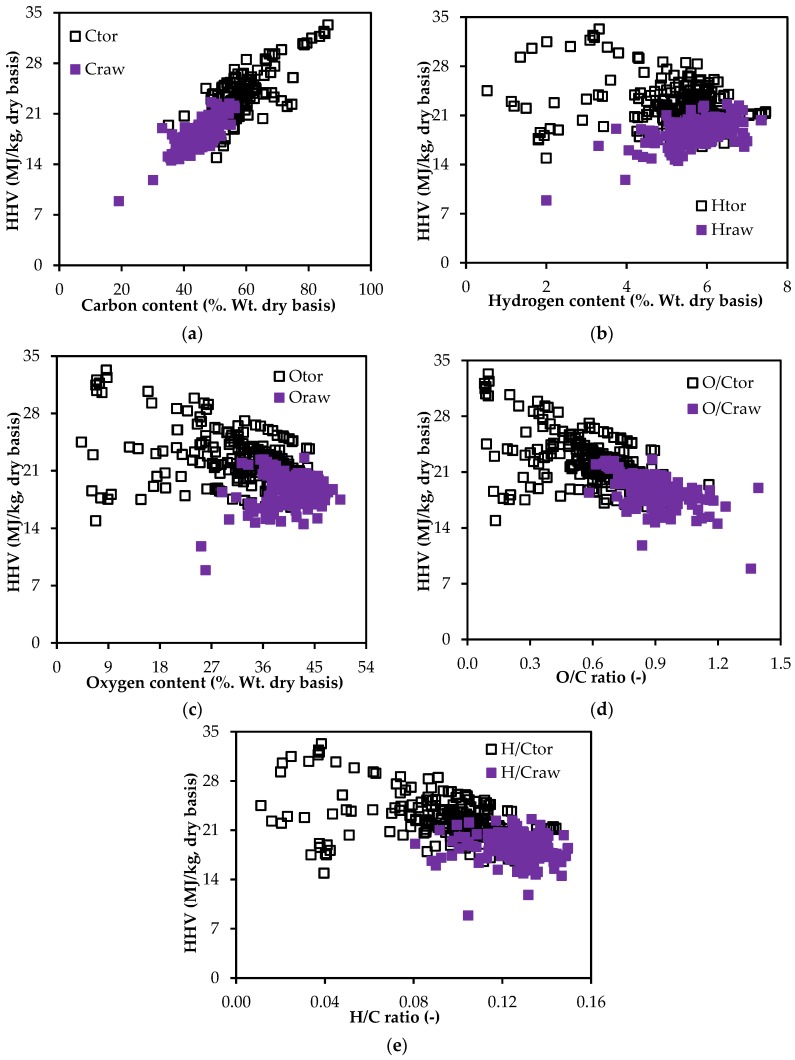
Variation of the HHV values with the compositions of ultimate analysis: (**a**) carbon; (**b**) hydrogen; (**c**) oxygen; (**d**) O/C; and (**e**) H/C.

Figure 1 and Figure 2 show how the HHV of biomass varies with different components of the proximate and ultimate analyses, respectively. Figure 1a indicates that the variation of HHV with the volatile matter content (VM) of raw biomass and of torrefied biomass has the opposite trend. While HHV of the torrefied biomass decreases with the increase in volatile matter content, the HHV of raw biomass increases with the VM. This tells us that the predicted HHV value of the torrefied biomass using the existing HHV correlations with a volatile matter term will have a high degree of uncertainty. On the other hand, Figure 1b,c show that the HHV values are in the same trend with the fixed carbon (FC) and ash (ASH) contents of both the torrefied and raw biomass. The scattered plot of the HHV with the FC shows that the HHVs of raw and torrefied biomass have a good trend and can be fitted to a single curve using FC as an independent variable. However, the variation of the HHV values with the ash content cannot be predicted by a single correlation. The existing correlations with the term ash content will underestimate the HHV of the torrefied biomass. Considering these facts, one can note that the HHV correlations based on the proximate analyses developed for the raw biomass have to be modified if they were to be used for torrefied biomass. Since the torrefaction process can affect all of the components of the proximate analysis, it is always good to have a new correlation with all components of the proximate analysis. This would help to incorporate all changes in the torrefied biomass.

The variations of the HHV of both the raw and torrefied biomass with respect to the compositions of the ultimate analysis are shown in Figure 2. Though the HHV values of the raw and torrefied biomass show a good relation with carbon content, hydrogen and oxygen contents have a more scattered distribution of the HHV values. The HHV of both raw and torrefied biomass increases with the carbon content, which agrees with the current studies [8,11]. While the HHV increases with the hydrogen content of raw biomass, it decreases in the torrefied biomass (Figure 2b). However, the HHV values decrease with the increase in the O/C and H/C ratios for both raw and torrefied biomass. Despite that a small change in nitrogen or sulfur contents may change the HHV, this study excluded variations of HHV with them because their concentrations in biomass materials were very small (Tables S1 and S2).

In addition to this, one can see from Figure 1 and Figure 2 that the variance of data points for the torrefied biomass is more compared to that for the raw biomass. One may argue that the trend should be the opposite as the torrefaction process degrades raw biomass and produces a more homogenized product. However, one should also need to consider that the data points of torrefied biomass used for plotting Figure 1 and Figure 2 have a wide variety of torrefied products produced from the different types of torrefaction processes, including dry torrefaction, wet torrefaction and pressurized torrefaction. The product qualities also depend on the operating conditions (temperature and time). The degree of degradation of raw biomass varies heavily based on the temperature of torrefaction and holding time. Additionally, the product qualities are also affected by the type of reactors (fixed bed, rotary reactor and fluidized bed reactors). Considering these, the large variances found in Figure 1 and Figure 2 for torrefied biomass are reasonable.

### 3.2. Validation of Existing Correlations Using Data from Torrefied Biomass

From Figure 1 and Figure 2, it is clear that existing correlations with individual proximate and ultimate analyses terms cannot be used to estimate the HHV of the torrefied biomass. To determine the possibility of using existing correlations, estimation errors for the selected existing correlations were calculated using the set of data presented in Table S1. Table 2 and Table 3 present the estimation errors for the existing HHV correlations based on proximate and ultimate analyses of torrefied biomass, respectively.

Proximate analysis-based correlations have more estimation errors compared to the ultimate analysis-based correlations. Moreover, one can confirm that the average biased error (ABE) was found to be negative for all reported correlations based on the proximate analysis. This confirms that the existing proximate analyses-based HHV correlations underestimate the higher heating value of torrefied biomass. The best correlation among the tested ultimate analysis-based correlations was the correlation presented (bolded in Table 3) by Friedl et al. [68]. However, given the fact that there are significant disagreements between the predicted and measured HHV values of torrefied biomass, the authors emphasize to the readers of this paper that they need be very cautious before using the existing HHV correlations. If they need to be used, it is encouraged to use the reported data from Table S1 for the validation.

### 3.3. New HHV Predicting Correlations

As the range of HHV and compositions (proximate and ultimate analyses) of torrefied biomass are changed significantly, it is essential to find the new correlations for predicting the HHV, which is applicable mainly for the torrefied biomass. This section provides the probable estimation errors of different possible forms of the new correlations presented in Table 1. The estimation errors were calculated by using the data points from Table S1. Correlations with the low MAE values could be used for predicting the HHV of the torrefied biomass. Table 4 and Table 5 present the summaries of the estimation errors calculated for the studied new HHV correlations based on the proximate analysis and the ultimate analysis, respectively. The total data points of torrefied biomass used was 246. The ranges of reported data for HHV, VM, FC, ASH, C, H and O were 14.90 MJ/kg–33.30 MJ/kg, 13.30%–88.57%, 11.25%–82.74%, 0.08%–47.62%, 35.08%–86.28%, 0.53%–7.46% and 4.31%–44.70%, respectively.

Among the studied new forms of proximate analysis-based HHV correlations, PSP4 (present study proximate), PSP11, and PSP12 (bolded in Table 4) have the lowest MAE values. Therefore, they can be used to predict the HHV of torrefied biomass materials with a good accuracy. Considering the ABE values, PSP4 (bold and italic in Table 4) shows the lowest ABE value. It is thus selected for future use. However, the predicted values, because of the positive ABE value of 0.60, will be slightly higher than the actual HHV values of torrefied biomass.

Among 16 new forms of ultimate analysis-based HHV correlations, the correlation PSU16 (present study ultimate; bold and italic in Table 5) has the lowest MAE value. It could, therefore, be deployed to predict the HHV of torrefied biomass. However, it also predicts a slightly higher HHV value than the actual HHV of torrefied biomass as the bolded correlation also has a small positive ABE value of 0.49.

Comparing the estimation errors in Table 4 and Table 5, one can see that the MAE values in Table 5 are much smaller than those values presented in Table 4. This confirms that the proximate analysis-based correlations will have more prediction error compared that of the ultimate analysis-based correlations. Therefore, one should be very careful to use proximate analysis-based HHV correlation to predict the HHV of torrefied biomass.

Despite that identical terms were used in determining the coefficients, PSU16 has a very different coefficient as compared to the correlation presented by Friedl et al. [68]. This may be due to a wide variety of data used, which will allow having more than one optimal solution in a multivariate regression process [68]. In addition to this, the authors also emphasize here that one should not be confused with the sign of the coefficient terms of the selected new correlations. The negative constant term for FC of the selected correlation (PSP4) does not represent the actual relation between HHV and FC (Figure 1a). Similarly, the negative coefficient of C-terms in the selected correlation (PSU16) does not represent the actual relation between HHV and C (Figure 2a).

The direct relationship between HHV and individual components of proximate and ultimate analyses as shown in Figure 1a–c and Figure 2a–c can be explained from developed correlations PSP (1–3) in Table 4 and PSU (1–3) in Table 5, respectively. The HHV of torrefied biomass increases with the increase in FC content, but decreases at high volatile matter and ash contents. Similarly, the HHV of torrefied biomass increases with the increase in carbon content, but decreases at high hydrogen and oxygen contents.

### 3.4. Validation of the Selected New Correlations

**Figure 3:**
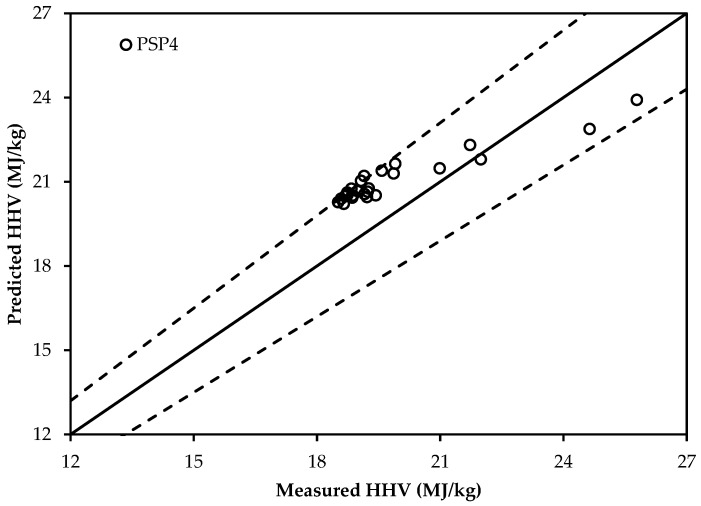
Validation of the selected proximate analysis-based correlation: PSP4 (error ±10%).

**Figure 4:**
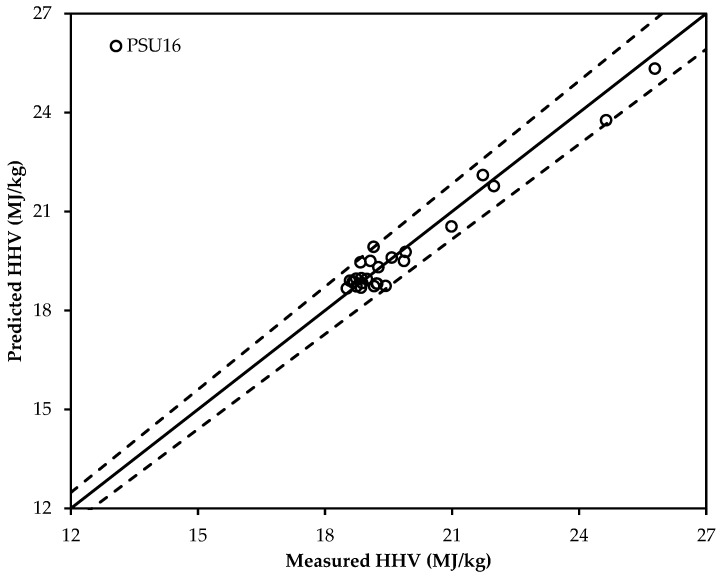
Validation of the selected ultimate analysis-based correlation: PSU16 (error ±4%).

Another set of data of torrefied biomass in Table 6 (26 samples) has been adopted to validate the selected new correlations. Figure 3 and Figure 4 show the deviation of the predicted and measured HHV values using the newly-selected proximate analysis-based correlation (PSP4) and the newly-selected ultimate analysis-based correlation (PSU16), respectively. From Figure 3 and Figure 4, it is confirmed that the ultimate analysis-based correlation has a better prediction compared that to the proximate-based correlation. Two additional lines of ±10% for Figure 3 and ±4% for Figure 4 are also shown, representing the percentage error of prediction. The residual distribution in Figure 3 does not look to be a perfectly normal distribution. Though there are few residuals on the negative side, most of the residuals are concentrated towards the positive side. This may be due to the positive ABE value that was found for the selected new HHV correlations. This can be supported from Figure 4, which shows how the residuals are distributed around the centerline. As the ultimate analysis-based correlation has a smaller ABE value compared to the proximate analysis-based correlation, the residuals in Figure 4 are more normally distributed around the centerline than in Figure 3.

The authors would also like to emphasize here that though the selected empirical correlations are based on a wide range of torrefied biomass materials, they have only small fractions of nitrogen and sulfur contents. Thus, the selected correlations may produce high estimation errors if they were to be used to predict HHV of materials with high nitrogen and sulfur contents. In addition, the empirical correlation developed in this study does not account for how the torrefaction process is carried out. Though the information used in this study includes torrefied materials produced from various torrefaction technologies (dry, wet and pressurized torrefaction) at different operating conditions (time, temperature, particle size, working media, pressure, heating rate and material types), the selected empirical correlations may also lead to a greater prediction error if they needed to predict HHV of torrefied biomass produced from a new technology.

## 4. Conclusions

The torrefaction process changes the properties of biomass. The changes in the properties affect the existing HHV predicting correlations. Results showed that not all existing HHV correlations could be deployed to predict the HHV of torrefied biomass. Estimation errors of correlations based on the proximate analysis were found significantly higher compared to the ultimate analysis-based HHV correlations. New correlations were then determined using the least sum square error method in Microsoft Excel. Comparing the MAE, AAE and ABE, new correlations with the least MAE value are selected to predict the HHV of torrefied biomass. The newly-selected correlations for predicting the HHV of torrefied biomass are:

HHV=0.1846VM+0.3525FC

$$HHV=\;32.7934+0.0053C^{2}-0.5321C-2.8769H+0.0608CH-0.2401N\;$$

The newly-selected correlations were then validated using another set of data (26 torrefied biomasses). They have good prediction accuracy within the error band of ±10% and are better than the existing correlations. Therefore, they could be used to predict the HHV of torrefied biomass.

These correlations would be of a great interest in the present context where research is growing on biomass torrefaction, and such correlations can help in reducing the cost of experimental tests and in saving testing time. The authors would like to declare here that though these new correlations could predict the HHV of torrefied biomass with a good accuracy, this paper does not indicate that the existing correlations are not suitable for predicting the HHV of raw biomass.

## Figures and Tables

**Table 1:** Studied new forms of higher heating value (HHV) correlations using proximate and ultimate analyses. PSP, present study proximate; PSU, present study ultimate.

Representation	New Forms of Correlations
	*Proximate analysis*
PSP1	HHV=a+b×ASH
PSP2	HHV=a+b×FC
PSP3	HHV=a+b×VM
PSP4	HHV=a×VM+b×FC
PSP5	HHV=a×FC+b×ASH
PSP6	HHV=a×ASH+b×VM
PSP7	HHV=a+b×VM+c×FC
PSP8	HHV=a+b×FC/VM
PSP9	$HHV=a+b\times FC+c\times FC^{2}$
PSP10	$HHV=a+b\times VM+c\times FC+d\times VM^{2}+e\times FC^{2}$
PSP11	$HHV=a+b\times VM+c\times ASH+d\times VM^{2}+e\times ASH^{2}$
PSP12	$HHV=a+b\times FC+c\times ASH+d\times FC^{2}+e\times ASH^{2}$
	*Ultimate analysis*
PSU1	HHV=a+b×C
PSU2	HHV=a+b×H
PSU3	HHV=a+b×O
PSU4	HHV=a+b×C+c×H
PSU5	HHV=a+b×C+c×O
PSU6	HHV=a+b×H+c×O
PSU7	HHV=a+b×C+c×H+d×N+e×O
PSU8	HHV=a+b×O/C
PSU9	HHV=a+b×H/C
PSU10	HHV=a+b×O/C+c×H/C
PSU11	$HHV=a+b\times C+c\times C^{2}$
PSU12	$HHV=a+b\times C+c\times H+d\times C^{2}+e\times H^{2}$
PSU13	$HHV=a+b\times C+c\times O+d\times C^{2}+e\times O^{2}$
PSU14	$HHV=a+b\times O/C+c\times {\left(O/C\right)}^{2}$
PSU15	$HHV=a+b\times O/C+c\times H/C+d\times {\left(O/C\right)}^{2}+e\times {\left(H/C\right)}^{2}$
PSU16	$HHV=a+b\times C^{2}+c\times C+d\times H+e\times CH+f\times N$

**Table 2:** Estimation errors of existing proximate analysis-based correlations using the properties of the torrefied biomass. VM, volatile matter; FC, fixed carbon; AAE, average absolute error; ABE, average bias error.

Equation (P)	Existing Proximate Analysis-Based Correlations	MAE	AAE	ABE	Ref.
1	HHV = 20.067 − 0.234ASH	3.67	15.48	−15.14	[61]
2	HHV = 26.601 − 0.304ASH − 0.082VM	2.89	12.26	−11.80	[61]
3	HHV = −10.81408 + 0.3133(FC + VM)	3.70	15.81	−15.29	[5]
4	HHV = 0.196FC + 14.119	3.03	13.37	−10.43	[4]
5	HHV = 0.312FC + 0.1534VM	3.33	14.43	−13.94	[4]
**6**	**HHV = 0.3543FC + 0.1708VM**	**1.58**	**6.88**	**−3.29**	**[6]**
7	HHV = 0.356248VM − 6.998497	6.85	28.94	−25.56	[62]
8	HHV = −0.0066FC^2^ + 0.5866FC + 8.752	3.66	15.50	−13.28	[63]
9	HHV = −0.0066VM^2^ + 0.7371VM + 1.2305	3.62	15.26	−12.08	[63]
10	HHV = 19.914 − 0.2324ASH	3.80	16.08	−15.79	[8]
11	HHV = −3.036 + 0.2218VM + 0.2601FC	3.39	14.36	−14.06	[8]
12	HHV = 0.3536FC + 0.1559VM − 0.0078ASH	2.25	9.82	−8.04	[7]
13	HHV = −0.1882VM + 32.94	3.24	14.68	−6.23	[64]
14	HHV = 0.1905VM + 0.2521FC	2.69	10.61	−10.61	[9]
15	HHV = 20.86 − 0.261ASH	3.14	13.19	−12.28	[65]
16	HHV = −13.173 + 0.416VM	8.75	37.27	−35.84	[65]
17	HHV = −2.057 − 0.092ASH + 0.279VM	6.99	29.71	−28.66	[65]
18	HHV = 35.4879 − 0.3023ASH − 0.1905VM	1.73	7.58	−3.68	[12]
19	HHV = 19.2880 − 0.2135VM/FC − 1.9584ASH/VM + 0.0234FC/ASH	3.40	14.19	−12.82	[1]
20	HHV = 18.96016 − 0.22527ASH	4.69	20.06	−19.94	[66]

**Table 3:** Estimation errors of existing ultimate analysis-based correlations using the properties of the torrefied biomass.

Equation (U)	Existing Ultimate Analysis-Based Correlations	MAE	AAE	ABE	Ref.
1	HHV = −3.147 + 0.468C	1.49	6.66	3.26	[65]
2	HHV = −1.642 − 0.024ASH + 0.475(C + N) − 0.376(H + N)	1.58	7.00	2.52	[65]
3	HHV = 23.668 − 7.032H − 0.002A^2^ + 0.005C^2^ + 0.771H^2^ + 0.019N^2^	2.95	12.93	11.11	[65]
4	HHV = −0.763 + 0.301C + 0.525H + 0.064O	1.73	7.24	−5.78	[61]
5	HHV = −1.3675 + 0.3137C + 0.7009H + 0.0318O	1.71	7.20	−5.96	[8]
6	HHV = 0.335C + 1.423H − 0.154O − 0.145N	1.59	6.99	5.35	[4]
7	HHV = 0.3259C + 3.4597	1.37	5.96	−2.37	[8]
8	HHV = 0.4373C − 1.6701	1.37	6.13	2.27	[67]
**9**	**HHV = (3.55C^2^ − 232C − 2230H + 51.2CH + 131N + 20600)×10^−3^**	**1.09**	**4.81**	**−0.52**	**[68]**
10	HHV = 0.879C + 0.3214H + 0.056O − 24.826	5.51	25.43	23.88	[11]
11	HHV = 0.924C − 22.403	7.14	31.10	30.19	[11]

**Table 4:** Comparison of the estimation errors of the developed correlations using proximate analysis of torrefied biomass (PSP, present study proximate analysis-based correlation).

Equation (P)	Developed Proximate Analysis-Based Correlations and Estimation Errors	MAE	AAE	ABE	Ref.
1	HHV = 22.9976 − 0.1135ASH	2.14	9.37	1.53	PSP1
2	HHV = 18.1418 + 0.1438FC	1.78	8.17	1.14	PSP2
3	HHV = 26.2841 − 0.0604VM	2.11	9.59	1.61	PSP3
***4***	***HHV = 0.1846VM + 0.3525FC***	***1.38***	***6.17***	***0.60***	***PSP4***
5	HHV = 0.6663FC − 0.0575ASH	6.60	29.64	−15.56	PSP5
6	HHV = 0.3545ASH + 0.2960VM	4.39	18.73	−1.88	PSP6
7	HHV = 2.4830 + 0.1602VM + 0.3225FC	1.40	6.25	0.75	PSP7
8	HHV = 21.1811 + 1.8812FC/VM	1.96	9.00	1.44	PSP8
9	HHV = 20.4755 + 0.0007FC + 0.0018FC^2^	1.73	7.97	1.12	PSP9
10	HHV = 3.7950 − 0.2177VM − 0.4096FC + 0.0011VM^2^ − 0.0004FC^2^	1.39	6.24	0.73	PSP10
11	HHV = 36.4042 − 0.2177VM − 0.4096ASH + 0.0005VM^2^ + 0.0023ASH^2^	1.38	6.21	0.72	PSP11
12	HHV = 19.5785 + 0.1111FC − 0.2602ASH + 0.0007FC^2^ + 0.0030ASH^2^	1.37	6.17	0.72	PSP12

**Table 5:** Comparison of the estimation errors of the developed correlations using ultimate analysis of torrefied biomass (PSU, present study ultimate analysis-based correlation).

Equation (U)	Developed Ultimate Analysis-based Correlations and Estimation Errors	MAE	AAE	ABE	Ref.
1	HHV = 4.4804 + 0.3194C	1.25	5.66	0.64	PSU1
2	HHV = 24.7975 − 0.4680H	2.23	10.01	1.72	PSU2
3	HHV = 26.5113 − 0.1278O	2.08	9.44	1.57	PSU3
4	HHV = 1.4036 + 0.3409C + 0.3586H	1.21	5.43	0.59	PSU4
5	HHV = 2.4544 + 0.3381C + 0.0300O	1.23	5.52	0.62	PSU5
6	HHV = 25.0602 + 0.9092H − 0.2290O	2.03	9.22	1.49	PSU6
7	HHV = 3.6165 + 0.3181C + 0.6107H − 0.4380N − 0.0613O	1.21	5.44	0.58	PSU7
8	HHV = 27.0624 − 7.8378O/C	1.88	8.54	1.35	PSU8
9	HHV = 28.1442 − 50.0874H/C	2.04	9.24	1.46	PSU9
10	HHV = 26.8463 − 8.8867O/C + 8.8489H/C	1.87	8.52	1.35	PSU10
11	HHV = 5.1906 + 0.2957C − 0.0002C^2^	1.25	5.66	0.64	PSU11
12	HHV = 7.8546 + 0.1255C + 0.1563H + 0.0018C^2^ − 0.0320H^2^	1.21	5.44	0.59	PSU12
13	HHV = 3.3965 + 00.3359C − 0.0666O + 0.0001C^2^ + 0.0019O^2^	1.23	5.54	0.62	PSU13
14	HHV = 27.2908 − 8.8671O/C + 0.9733(O/C)^2^	1.88	8.55	1.36	PSU14
15	HHV = 25.5411 − 186247O/C + 103.1710H/C + 8.0136(O/C)^2^ − 515.0026(H/C)^2^	1.85	8.45	1.32	PSU15
***16***	***HHV = 32.7934 + 0.0053C^2^* − *0.5321C* − *2.8769H + 0.0608CH* − *0.2401N***	***1.13***	***5.01***	***0.49***	***PSU16***

**Table 6:** HHV, proximate analyses and ultimate analyses for model verification (dry basis).

Material	MJ/kg	Proximate Analysis (%)			Ultimate Analysis (%)					Ref.
	HHV	VM	FC	ASH	C	H	N	O	S	
Corn stover	18.59	75.38	18.39	6.23	45.88	5.90	0.50	41.52	0.05	[69]
	18.69	74.87	18.89	6.24	45.76	5.90	0.46	41.67	0.04	
	18.88	75.50	18.64	5.85	45.65	5.86	0.52	42.23	0.05	
	18.74	75.57	18.90	5.54	46.03	5.92	0.44	42.13	0.04	
	18.86	76.24	18.04	5.73	46.09	5.88	0.47	41.91	0.06	
	18.75	75.10	18.94	5.96	45.26	5.78	0.50	42.53	0.04	
	18.99	75.12	19.29	5.61	46.02	5.85	0.48	42.11	0.06	
	18.65	74.78	18.18	7.04	45.72	5.80	0.51	40.96	0.05	
	18.85	74.20	19.25	6.53	45.19	5.71	0.60	42.07	0.04	
	18.52	74.76	18.36	6.87	45.10	5.66	0.62	41.84	0.05	
	19.22	73.17	19.71	7.11	45.60	5.64	0.61	41.22	0.05	
	19.16	74.27	19.43	6.30	45.31	5.70	0.57	42.26	0.04	
	19.23	73.14	20.27	6.59	45.54	5.63	0.56	41.89	0.05	
	19.43	72.84	20.06	7.09	45.31	5.60	0.58	41.63	0.04	
	19.26	70.38	22.03	7.58	47.35	5.27	0.76	39.13	0.06	
	19.07	70.54	22.72	6.73	47.92	5.37	0.68	39.36	0.04	
	19.87	68.28	24.65	7.07	48.01	5.07	0.74	39.14	0.05	
	19.58	68.48	24.82	6.70	48.36	5.14	0.75	39.13	0.05	
	19.91	65.03	27.34	7.63	48.94	4.99	0.69	37.82	0.05	
Olive stones	20.99	77.40	20.40	2.20	50.30	6.50	0.30	40.10	0.00	[70]
	21.99	75.50	22.30	2.20	53.30	6.40	0.20	37.90	0.00	
	24.64	67.80	29.40	2.80	58.30	6.10	0.40	32.40	0.00	
	25.79	61.20	35.80	2.90	62.10	5.80	0.30	28.80	0.00	
Rape straw	18.84	72.33	20.99	6.67	47.23	5.22	0.00	41.19	0.00	[71]
Wheat straw	19.15	68.59	24.24	7.17	48.49	6.62	0.00	38.11	0.00	
	21.73	56.85	33.52	9.62	56.12	4.63	0.00	29.97	0.00	

## Supplementary Materials

The following are available online at http://www.mdpi.com/2306-5354/4/1/7/s1. Table S1: HHV, proximate analyses and ultimate analyses of torrefied biomass (dry basis); Table S2: HHV, proximate analyses and ultimate analyses of raw biomass (dry basis).
