# Supplementary material for: HHV Predicting Correlations for Torrefied Biomass Using Proximate and Ultimate Analyses
Source: Bioengineering (Basel). 2017 Jan 24;4(1):7. doi: 10.3390/bioengineering4010007 (PMC5590445; doi:10.3390/bioengineering4010007)
Supplement: Supplementary file 1 [file bioengineering-04-00007-s001.pdf]

Supplementary Materials: HHV Predicting Correlations for Torrefied Biomass Using Proximate and Ultimate Analyses

Daya Ram Nhuchhen and Muhammad T. Afzal

Table S1. HHV, proximate analyses and ultimate analyses of torrefied biomass (dry basis).

Material	MJ/kg	Proximate Analysis (%)			Ultimate Analysis (%)					Ref.
	HHV	VM	FC	ASH	C	H	N	O	S	
Acacia mangium (hardwood)	31.70	14.92	80.05	5.03	83.58	3.09	0.84	7.45	0.01	[19]
Acacia mangium (hardwood)	21.90	70.06	27.47	2.47	54.95	5.47	0.60	36.47	0.04	[19]
Ash wood	20.00	74.00	24.10	1.90	51.90	5.70	0.25	40.20	0.00	[20]
Ash wood	21.00	66.20	31.70	2.10	55.40	5.50	0.25	36.80	0.00	[20]
Ash wood	24.20	51.90	45.60	2.50	62.10	5.10	0.30	30.10	0.00	[20]
Birch	20.21	88.57	11.34	0.09	49.42	6.38	0.12	44.07	0.02	[21]
Birch	20.01	87.97	11.94	0.08	49.61	6.16	0.13	44.10	0.02	[21]
Birch	20.78	85.15	14.76	0.09	51.25	6.18	0.11	42.46	0.02	[21]
Birch	20.51	82.64	17.27	0.10	51.34	5.94	0.13	42.59	0.02	[21]
Birch	22.93	73.78	26.09	0.13	56.92	5.86	0.09	37.13	0.02	[21]
Birch	23.76	88.19	11.25	0.56	49.56	6.07	0.18	43.63	0.00	[22]
Birch	24.57	83.40	16.04	0.55	52.19	5.95	0.23	41.08	0.00	[22]
Birch	25.33	79.67	19.68	0.65	54.87	5.76	0.23	38.49	0.00	[22]
Birch	23.68	86.67	12.99	0.34	49.21	6.11	0.22	44.12	0.00	[22]
Birch	24.67	81.37	18.41	0.22	52.29	6.00	0.26	41.24	0.00	[22]
Birch	25.08	75.47	24.28	0.25	54.02	5.82	0.26	39.65	0.00	[22]
Camphorwood	18.90	82.62	16.21	1.17	50.42	6.12	0.41	41.87	0.00	[23]
Camphorwood	20.45	79.20	19.36	1.44	52.29	5.97	0.30	40.02	0.00	[23]
Camphorwood	22.35	70.91	26.55	2.54	60.37	4.89	0.22	31.98	0.00	[23]
Cassava rhizome	18.50	70.40	18.90	10.70	46.30	5.60	0.88	36.40	0.09	[12]
Cassava rhizome	19.18	68.70	20.80	10.50	49.30	5.10	0.96	34.00	0.09	[12]
Cassava rhizome	19.65	64.70	23.10	12.20	49.10	5.10	0.94	32.60	0.09	[12]
Cassava rhizome	19.79	65.80	22.60	11.60	50.60	5.20	0.97	31.50	0.09	[12]
Cassava rhizome	21.12	58.70	29.50	11.80	52.50	4.50	1.10	30.00	0.09	[12]
Cedar wood	19.87	81.96	17.12	0.92	54.24	5.71	0.28	38.86	0.00	[23]
Cedar wood	20.61	79.25	18.76	1.99	52.35	5.90	0.25	39.53	0.00	[23]
Cedar wood	23.17	65.34	32.64	2.02	59.79	5.17	0.22	32.81	0.00	[23]
Corncob	19.01	74.70	19.30	6.00	48.20	5.40	0.94	39.40	0.08	[12]
Corncob	21.28	64.20	28.30	7.50	54.50	5.50	1.20	31.20	0.10	[12]
Corncob	21.45	61.90	29.90	8.20	53.40	4.90	1.20	32.20	0.11	[12]
Corncob	23.83	49.40	40.00	10.60	62.30	4.60	1.50	20.90	0.10	[12]
Cotton stalk	19.30	74.00	19.50	6.50	47.87	5.84	0.94	38.71	0.19	[24]
Cotton stalk	19.50	72.50	20.50	7.00	49.01	5.63	1.09	37.11	0.22	[24]
Cotton stalk	19.40	72.00	21.20	6.80	49.49	5.66	1.05	36.72	0.25	[24]
Cotton stalk	21.00	70.00	22.00	8.00	50.88	5.67	1.27	33.95	0.25	[24]
Cotton stalk	20.70	64.00	27.00	9.00	51.96	5.55	1.19	31.94	0.28	[24]
Cotton stalk	21.90	58.50	30.00	11.50	54.52	5.14	1.35	27.17	0.32	[24]
Cotton stalk	22.00	62.00	28.00	10.00	53.28	5.28	1.28	29.79	0.30	[24]
Cotton stalk	23.30	53.00	32.00	15.00	54.23	4.95	1.47	24.06	0.33	[24]
Cotton stalk	23.10	49.00	38.00	13.00	62.64	4.91	1.77	17.31	0.39	[24]
Cotton stalk (dried)	17.09	74.25	18.56	7.19	51.92	5.95	0.91	34.03	0.00	[25]
Cotton stalk (dried)	18.85	69.76	20.32	9.92	56.18	5.43	0.83	27.64	0.00	[25]
Cotton stalk (dried)	20.31	56.23	32.49	11.28	65.35	4.92	0.96	17.49	0.00	[25]
Cotton stalk briquette-bottom	30.54	30.85	59.00	10.15	78.66	1.64	1.11	7.91	0.54	[26]
Cotton stalk briquette-center	31.47	23.91	67.23	8.87	81.08	2.01	0.75	6.81	0.47	[26]
Cotton stalk briquette-top	30.79	39.73	52.25	8.02	79.52	2.60	2.34	6.97	0.63	[26]
Cotton stalk particle	29.28	22.32	63.41	14.27	67.43	1.35	0.09	16.56	0.30	[26]
Eucalyptus	22.44	66.81	31.59	1.60	56.88	5.90	0.00	35.62	0.00	[27]
Eucalyptus	23.81	69.78	28.22	2.00	60.66	5.68	0.00	31.65	0.00	[27]
Eucalyptus	26.70	58.97	38.83	2.20	67.87	5.18	0.00	24.55	0.00	[27]
Eucalyptus	20.60	75.00	24.70	0.30	57.80	4.90	0.00	37.00	0.00	[28]
Eucalyptus	21.50	70.90	28.90	0.20	59.70	4.70	0.00	35.40	0.00	[28]

Eucalyptus	23.90	55.60	44.10	0.30	68.20	4.20	0.00	27.30	0.00	[28]
Eucalyptus	26.00	41.90	57.90	0.40	75.00	3.60	0.00	21.00	0.00	[28]
Eucalyptus	26.00	38.90	62.00	0.10	75.10	3.60	0.00	21.10	0.00	[28]
Eucalyptus	20.80	66.00	33.80	0.20	60.60	4.20	0.00	35.00	0.00	[28]
Eucalyptus	23.30	49.50	50.20	0.30	69.00	3.00	0.00	27.70	0.00	[28]
Eucalyptus	22.80	43.80	55.90	0.30	71.30	2.20	0.00	26.30	0.00	[28]
Eucalyptus	22.00	41.70	57.80	0.30	73.20	1.50	0.00	25.00	0.00	[28]
Eucalyptus	22.30	38.80	60.70	0.40	74.70	1.20	0.00	23.70	0.00	[28]
Eucalyptus	19.60	80.50	19.00	0.50	48.95	6.17	0.20	44.08	0.00	[29]
Eucalyptus	20.10	78.70	20.80	0.50	50.94	5.87	0.10	42.49	0.00	[29]
Eucalyptus	22.20	75.40	23.90	0.70	52.73	6.06	0.10	40.32	0.00	[29]
Eucalyptus	21.80	74.50	24.50	1.00	52.47	5.84	0.10	40.49	0.00	[29]
Eucalyptus	22.50	73.30	25.80	0.90	53.71	5.85	0.10	39.34	0.00	[29]
Eucalyptus	21.90	74.10	24.90	1.00	53.26	5.94	0.10	39.60	0.00	[29]
Eucalyptus	19.70	79.10	20.10	0.80	49.60	5.95	0.10	43.45	0.00	[29]
Eucalyptus	20.60	72.90	26.20	0.90	54.31	5.75	0.20	38.75	0.00	[29]
Eucalyptus	22.70	69.60	29.20	1.20	55.03	5.73	0.10	37.84	0.00	[29]
Eucalyptus	22.80	69.20	30.40	0.40	55.18	5.78	0.10	38.45	0.00	[29]
Eucalyptus	22.90	69.50	29.60	0.90	55.69	5.85	0.20	37.26	0.00	[29]
Eucalyptus	23.30	68.50	30.30	1.20	56.12	5.83	0.20	36.46	0.00	[29]
Eucalyptus	20.80	76.00	23.20	0.80	57.34	5.85	0.10	36.11	0.00	[29]
Eucalyptus	22.80	69.00	30.10	0.90	57.28	5.55	0.10	36.07	0.00	[29]
Eucalyptus	23.40	67.00	31.90	1.10	57.16	5.44	0.20	35.90	0.00	[29]
Eucalyptus	25.00	61.50	36.90	1.60	62.48	5.22	0.20	30.41	0.00	[29]
Eucalyptus	25.20	59.60	39.10	1.30	61.29	5.43	0.20	31.68	0.00	[29]
Eucalyptus saligna (hardwood)	22.70	73.77	25.34	0.89	55.41	5.73	0.45	37.48	0.04	[19]
Eucalyptus saligna (hardwood)	32.40	16.52	81.04	2.44	85.02	3.16	0.57	8.80	0.01	[19]
Grape pomace	24.32	69.30	29.20	1.40	57.82	6.12	2.16	32.49	0.00	[30]
Grape pomace	24.84	67.70	31.30	1.10	59.50	6.04	1.70	31.66	0.00	[30]
Grape pomace	25.75	67.10	30.90	1.90	60.22	6.28	1.69	29.91	0.00	[30]
Grape pomace	25.65	64.60	33.70	1.70	60.82	6.04	1.76	29.69	0.00	[30]
Grape pomace	26.10	64.60	33.20	2.10	61.73	6.18	1.83	28.17	0.00	[30]
Grape pomace	27.03	58.60	37.90	3.40	63.65	5.60	2.10	25.24	0.00	[30]
Grape pomace	28.31	54.30	43.00	2.70	66.48	5.76	2.26	22.82	0.00	[30]
Grape pomace	23.12	63.90	27.80	8.20	51.33	5.83	2.02	32.59	0.00	[30]
Grape pomace	25.29	51.20	38.20	10.50	56.30	4.84	2.37	25.99	0.00	[30]
Hardwood (mixture of oak and birch)	22.97	71.48	27.52	1.00	57.12	6.04	0.00	35.84	0.00	[27]
Hardwood (mixture of oak and birch)	22.63	70.85	27.55	1.60	57.47	5.61	0.00	35.23	0.00	[27]
Hardwood (mixture of oak and birch)	23.79	63.24	34.66	2.10	60.11	5.58	0.00	32.21	0.00	[27]
Juniper	21.31	79.10	19.72	1.15	56.83	5.75	0.20	36.16	0.01	[31]
Juniper	21.12	78.08	20.69	1.21	56.75	5.77	0.18	36.05	0.02	[31]
Leucaena	21.09	86.90	12.60	0.50	51.44	7.36	0.70	40.00	0.00	[32]
Leucaena	21.27	84.90	14.50	0.60	51.69	7.46	0.70	39.56	0.00	[32]
Leucaena	21.49	84.10	15.40	0.50	52.24	7.46	0.70	39.10	0.00	[32]
Leucaena	21.47	83.70	15.70	0.60	52.09	7.46	0.70	39.16	0.00	[32]
Leucaena	21.19	83.00	16.00	1.00	51.68	7.33	0.69	39.30	0.00	[32]
Leucaena	21.24	83.00	15.80	1.20	51.77	7.31	0.69	38.73	0.00	[32]
Leucaena	23.39	79.60	19.50	0.90	56.69	6.34	0.79	35.28	0.00	[32]
Leucaena	23.71	76.10	22.70	1.20	57.21	6.42	0.79	34.38	0.00	[32]
Leucaena	23.98	77.60	21.50	0.90	57.68	6.54	0.79	34.09	0.00	[32]
Leucaena	23.83	77.30	21.60	1.10	57.46	6.43	0.79	34.22	0.00	[32]
Leucaena	21.66	81.60	17.30	1.10	53.41	6.33	0.69	38.47	0.00	[32]
Leucaena	23.19	71.30	27.80	0.90	57.18	5.65	0.89	35.38	0.00	[32]
Leucaena	23.89	67.50	30.80	1.70	58.19	5.70	0.88	33.52	0.00	[32]
Leucaena	23.94	66.20	32.30	1.50	58.71	5.52	0.89	33.39	0.00	[32]
Leucaena	25.34	60.00	38.20	1.80	61.18	5.50	0.98	30.54	0.00	[32]
Leucaena	21.00	85.30	14.00	0.70	51.34	7.05	0.70	40.51	0.00	[33]
Leucaena	21.20	84.30	14.90	0.80	51.98	7.04	0.76	39.48	0.00	[33]
Leucaena	21.20	82.20	16.90	0.90	52.52	6.34	0.69	39.54	0.00	[33]
Leucaena	22.80	73.80	24.90	1.30	56.46	5.43	0.79	36.03	0.00	[33]
Leucaena	21.20	76.60	22.10	1.30	53.10	5.53	0.79	39.28	0.00	[33]
Leucaena	23.30	69.80	28.90	1.40	57.78	5.13	0.99	34.71	0.00	[33]

Leucaena	24.40	61.80	36.70	1.50	60.38	5.32	0.99	31.82	0.00	[33]
Loblolly pine chips	22.70	78.60	20.80	0.60	55.00	5.94	0.11	38.30	0.00	[34]
Loblolly pine chips	24.00	76.40	22.80	0.80	57.30	5.79	0.14	36.00	0.00	[34]
Loblolly pine chips	26.30	59.90	38.60	1.40	65.80	4.87	0.28	27.60	0.00	[34]
Mesquite	20.38	74.29	23.20	2.45	55.69	5.61	0.80	35.42	0.04	[31]
Mesquite	20.81	73.03	24.49	2.51	56.12	5.60	0.85	34.89	0.05	[31]
Miscanthus	18.90	72.80	23.80	3.40	49.20	5.80	0.25	41.40	0.00	[20]
Miscanthus	21.30	62.70	33.20	4.00	54.60	5.50	0.30	35.60	0.00	[20]
Miscanthus	24.60	46.50	47.70	5.80	63.00	5.00	0.35	26.00	0.00	[20]
Miscanthus	19.90	83.30	15.70	0.50	48.80	5.90	0.20	44.70	0.00	[35]
Miscanthus	21.40	81.90	17.50	0.70	49.60	5.90	0.30	43.80	0.00	[35]
Miscanthus	25.70	68.90	30.30	0.90	61.20	5.30	0.40	32.60	0.00	[35]
Miscanthus	20.30	84.80	14.20	0.90	49.60	5.70	0.10	43.20	0.00	[35]
Miscanthus A	21.60	65.24	33.33	1.43	55.00	5.72	0.00	37.85	0.00	[36]
Miscanthus B	20.60	78.20	20.47	1.33	52.99	5.92	0.00	39.76	0.00	[36]
Miscanthus D	24.50	61.60	36.45	1.95	62.16	5.59	0.00	30.30	0.00	[36]
Miscanthus giganteus	19.10	82.30	13.35	4.35	46.56	5.86	0.35	42.87	0.00	[37]
Miscanthus giganteus	19.37	81.74	13.37	4.90	47.36	5.73	0.44	41.58	0.00	[37]
Miscanthus giganteus	20.43	81.43	12.69	5.88	47.51	5.40	0.45	40.75	0.00	[37]
Miscanthus giganteus	19.45	80.35	14.79	4.86	49.28	5.27	0.50	40.09	0.00	[37]
Miscanthus giganteus	21.81	79.84	14.41	5.75	49.84	5.22	0.59	38.59	0.00	[37]
Miscanthus giganteus	21.57	64.87	27.23	7.90	52.88	4.55	0.55	34.12	0.00	[37]
Oil palm fibre	20.10	63.90	27.30	8.90	52.80	5.10	1.30	31.70	0.20	[28]
Oil palm fibre	21.40	56.60	32.40	11.00	54.90	5.00	1.50	27.50	0.10	[28]
Oil palm fibre	23.40	41.30	44.40	14.30	61.30	4.30	1.40	18.50	0.20	[28]
Oil palm fibre	23.70	32.80	52.40	14.80	65.50	3.40	1.90	14.20	0.20	[28]
Oil palm fibre	23.90	29.10	55.30	15.50	66.40	3.30	1.80	12.70	0.10	[28]
Oil palm fibre	20.30	39.20	44.80	16.00	56.90	2.90	2.30	21.70	0.20	[28]
Oil palm fibre	18.90	33.90	46.20	19.90	56.00	2.30	2.50	19.00	0.20	[28]
Oil palm fibre	19.10	32.30	45.70	22.10	55.90	2.10	2.70	16.90	0.20	[28]
Oil palm fibre	17.50	29.40	44.10	26.60	53.30	1.80	3.30	14.70	0.30	[28]
Oil palm fibre	14.90	25.30	38.40	36.40	50.50	2.00	4.10	6.80	0.20	[28]
Oil palm frond	20.20	70.90	24.70	4.50	52.70	5.60	0.26	36.90	0.07	[12]
Oil palm frond	21.21	66.40	28.60	5.00	55.20	5.20	0.27	34.20	0.08	[12]
Oil palm frond	21.95	63.50	31.10	5.40	56.40	4.90	0.32	32.90	0.07	[12]
Oil palm frond	22.48	61.00	33.40	5.60	58.40	5.00	0.36	30.60	0.07	[12]
Oil palm frond	24.41	53.00	40.60	6.40	63.20	4.70	0.35	25.30	0.08	[12]
Olive stone (Olive europaea)	22.00	77.10	22.53	0.37	55.04	5.80	0.17	38.60	0.02	[19]
Olive stone (Olive europaea)	33.30	15.98	82.74	1.28	86.28	3.32	0.46	8.65	0.01	[19]
Olive stones	20.67	60.78	23.17	16.05	40.13	4.28	1.93	37.61	0.00	[38]
Palm kernel shells	21.53	67.40	24.80	7.80	54.31	5.07	0.46	32.36	0.00	[39]
Palm kernel shells	21.89	63.80	28.30	7.90	55.35	4.88	0.46	31.41	0.00	[39]
Palm kernel shells	24.18	51.10	39.40	9.50	60.64	4.34	0.54	24.98	0.00	[39]
Palm kernel shells	20.92	68.91	25.59	5.50	54.96	5.93	0.60	33.01	0.00	[40]
Palm kernel shells	21.38	61.45	31.73	6.82	55.70	5.10	0.60	31.78	0.00	[40]
Palm kernel shells	20.99	54.72	37.27	8.01	53.91	5.37	0.89	31.82	0.00	[40]
Palm kernel shells	21.77	51.11	40.24	8.65	56.31	5.56	0.78	28.70	0.00	[40]
Pine	19.60	79.10	20.90	0.40	51.40	6.00	0.10	42.50	0.00	[20]
Pine	20.60	74.00	25.50	0.50	54.60	5.80	0.10	39.10	0.00	[20]
Pine	22.70	65.30	34.20	0.50	59.50	5.50	0.10	34.50	0.00	[20]
Pine	20.48	83.87	16.02	0.11	52.22	5.18	0.55	41.94	0.00	[41]
Pine	22.23	75.66	24.16	0.18	57.33	4.95	0.37	37.17	0.00	[41]
Pine	22.54	69.53	30.21	0.26	59.03	4.78	0.34	35.59	0.00	[41]
Pine	29.10	46.23	53.32	0.44	68.43	4.31	0.35	26.47	0.00	[41]
Pine	29.28	45.10	54.41	0.49	69.20	4.28	0.32	25.71	0.00	[41]
Pine	29.87	35.56	63.94	0.50	71.39	3.80	0.31	24.00	0.00	[41]
Pine shells	23.52	70.10	27.88	2.02	53.30	5.39	0.39	38.90	0.00	[38]
Pinus illiotis (softwood)	21.80	73.29	25.85	0.86	57.11	5.76	0.48	35.77	0.02	[19]
Pinus illiotis (softwood)	30.70	25.72	72.56	1.72	78.24	3.52	0.60	15.92	0.01	[19]
Residue (*Eucalyptus saligna*)	22.50	69.86	28.11	2.03	57.04	5.64	0.67	34.58	0.04	[19]
Residue (*Eucalyptus saligna*)	32.10	15.66	80.73	3.61	85.31	3.20	0.89	6.97	0.02	[19]
Residues (Norwegian forest)	20.56	72.38	25.34	2.38	51.13	5.93	0.49	40.26	0.07	[42]
Residues (Norwegian forest)	22.91	64.31	32.84	2.85	56.84	5.51	0.61	34.29	0.06	[42]
Rice husk	16.63	65.05	19.87	15.09	42.38	5.33	0.57	36.45	0.18	[43]
Rice husk	16.92	60.78	23.03	16.19	44.17	5.42	0.69	33.36	0.17	[43]

Rice husk	17.53	54.83	27.64	17.54	46.07	4.86	0.70	30.68	0.15	[43]
Rice husk	17.95	40.06	37.68	22.26	50.15	4.32	0.71	22.38	0.18	[43]
Rice husk (raw charred)	17.68	14.17	40.78	45.05	44.73	1.80	0.73	7.69	0.00	[44]
Rice husk (torrefied and charred)	18.55	13.30	44.43	42.27	48.89	1.85	0.86	6.13	0.00	[44]
Rice husk (torrefied and washed)	18.53	63.44	17.12	19.44	45.86	5.89	0.40	28.40	0.02	[44]
Rice husk (Torrefied)	18.73	59.16	20.66	20.18	47.45	4.26	0.41	27.68	0.03	[44]
Rice husk (torrefied, washed and charred)	18.12	15.94	42.24	41.82	45.96	1.95	0.75	9.52	0.00	[44]
Rice husk (washed and charred)	17.52	15.19	40.81	44.00	44.44	1.81	0.83	8.92	0.00	[44]
Rice husk (washed)	17.03	71.68	12.85	15.47	39.93	6.43	0.30	37.83	0.04	[35]
Rice husk briquette	24.51	18.77	33.61	47.62	47.09	0.53	0.06	4.31	0.38	[26]
Rice husk particle	22.96	26.94	29.85	43.20	48.78	1.13	0.07	6.32	0.49	[26]
Rice straw	17.38	68.90	16.20	14.90	45.80	5.50	0.90	32.80	0.09	[12]
Rice straw	18.45	62.50	19.60	17.90	47.40	4.90	0.97	28.80	0.08	[12]
Rice straw	18.78	59.80	21.70	18.50	49.10	5.10	0.98	26.20	0.07	[12]
Rice straw	18.89	58.90	22.20	18.90	46.90	5.00	1.00	28.10	0.07	[12]
Rice straw	20.73	46.00	30.00	24.00	51.40	4.40	1.20	18.90	0.07	[12]
Rice straw	20.30	77.50	18.00	4.50	51.19	6.04	0.24	37.72	0.29	[25]
Rice straw	21.30	75.00	19.70	5.30	51.14	5.89	0.25	37.12	0.29	[24]
Rice straw	21.40	74.50	20.00	5.50	51.41	5.71	0.26	36.86	0.28	[24]
Rice straw	21.20	72.00	21.80	6.20	51.31	5.77	0.21	36.21	0.26	[24]
Rice straw	22.60	69.00	26.50	4.50	57.40	5.63	0.28	32.09	0.16	[24]
Rice straw	24.60	62.00	32.00	6.00	57.06	5.58	0.29	30.93	0.17	[24]
Rice straw	26.50	58.00	37.00	5.00	57.38	5.57	0.29	31.64	0.12	[24]
Rice straw	28.50	44.00	48.00	8.00	60.08	5.47	0.29	26.13	0.12	[24]
Rice straw	28.60	43.50	49.00	7.50	66.14	4.90	0.40	20.91	0.13	[24]
Softwood (mixture of spruce, pine and larch)	22.08	79.62	20.28	0.10	54.55	6.29	0.00	39.06	0.00	[27]
Softwood (mixture of spruce, pine and larch)	22.33	78.07	21.63	0.30	55.53	6.08	0.00	38.09	0.00	[27]
Softwood (mixture of spruce, pine and larch)	23.61	71.51	28.09	0.40	58.47	5.98	0.00	35.16	0.00	[27]
Spruce	22.05	75.65	24.15	0.20	55.33	5.73	0.09	38.80	0.00	[45]
Spruce	22.35	82.56	17.36	0.08	55.27	5.99	0.07	38.67	0.00	[45]
Spruce	22.64	81.51	18.39	0.10	55.75	6.05	0.06	38.14	0.00	[45]
Spruce	20.81	85.72	14.17	0.11	51.34	6.18	0.07	42.42	0.02	[21]
Spruce	21.02	84.64	15.22	0.14	51.21	6.39	0.06	42.35	0.02	[21]
Spruce	21.33	83.92	15.95	0.12	52.55	6.15	0.06	41.23	0.02	[21]
Spruce	21.51	81.87	18.04	0.09	53.69	5.89	0.06	40.36	0.02	[21]
Spruce	22.97	74.74	25.12	0.14	56.99	5.87	0.07	37.07	0.02	[21]
Spruce	25.26	82.40	17.06	0.54	53.79	6.03	0.14	39.51	0.00	[22]
Spruce	26.05	80.76	18.72	0.52	56.56	5.85	0.16	36.90	0.00	[22]
Spruce	26.59	74.82	24.68	0.50	59.11	5.52	0.17	34.70	0.00	[22]
Spruce	24.91	82.29	17.36	0.35	52.99	5.98	0.12	40.56	0.00	[22]
Spruce	25.89	78.79	21.00	0.21	56.12	5.85	0.14	37.69	0.00	[22]
Spruce	26.45	72.83	26.96	0.21	58.48	5.60	0.14	35.58	0.00	[22]
Spruce (Norwegian)	16.55	75.19	24.33	0.48	52.72	5.88	0.06	40.86	0.03	[42]
Spruce (Norwegian)	19.53	80.21	19.37	0.44	48.86	6.08	0.05	44.57	0.03	[42]
Spruce wood	20.59	84.00	15.70	0.30	51.40	5.90	0.10	42.30	0.00	[46]
Spruce wood	21.42	80.30	19.30	0.40	53.60	5.90	0.10	40.00	0.00	[46]
Spruce wood	22.14	77.50	22.10	0.40	55.20	5.70	0.10	38.60	0.00	[46]
Spruce wood	22.19	77.30	22.30	0.40	55.20	5.60	0.10	38.70	0.00	[46]
Spruce wood	22.33	76.20	23.40	0.40	55.80	5.80	0.10	37.90	0.00	[46]
Spruce wood	27.59	51.50	47.80	0.70	69.20	5.00	0.10	25.00	0.00	[46]
Sugar cane	18.76	73.80	18.60	7.60	47.90	5.60	0.49	38.20	0.18	[12]
Sugar cane	20.01	66.70	23.80	9.50	50.90	5.40	0.30	33.70	0.17	[12]
Sugar cane	20.01	67.40	23.00	9.60	51.70	5.20	0.34	33.00	0.21	[12]
Sugar cane	21.65	58.40	31.70	9.90	56.50	5.10	0.46	27.80	0.23	[12]
Switch grass	27.11	67.52	27.51	4.98	56.21	4.44	0.42	32.81	1.14	[47]
Switch grass	23.53	78.99	17.38	3.63	50.87	5.56	0.31	38.14	1.48	[47]
Switch grass (densified and torrefied)	22.27	62.63	31.45	5.91	49.01	4.83	0.37	38.90	0.99	[47]
Wheat straw	18.10	65.60	25.00	9.40	47.40	5.50	1.00	36.80	0.00	[20]

Wheat straw	20.70	51.80	35.90	12.40	52.90	5.00	1.20	28.50	0.00	[20]
Wheat straw	23.00	39.80	45.10	15.20	57.00	4.50	1.40	21.90	0.00	[20]
Wheat straw	19.40	55.50	24.22	20.28	35.08	3.43	0.64	40.58	0.00	[38]
Willow	22.09	73.43	26.07	0.50	55.32	5.97	0.00	38.21	0.00	[27]
Willow	22.64	71.89	27.41	0.70	56.50	5.86	0.00	37.04	0.00	[27]
Willow	23.93	62.50	36.40	1.10	59.44	5.74	0.00	33.82	0.00	[27]
Willow A	21.80	74.03	23.82	2.15	55.29	6.07	0.81	35.72	0.00	[36]
Willow B	21.00	77.43	21.04	1.53	53.47	5.91	0.75	38.30	0.00	[36]
Willow D	23.60	68.34	29.83	1.84	59.19	5.69	0.35	32.79	0.00	[36]

Table S2. HHV, proximate analyses and ultimate analyses of raw biomass (dry basis).

Material	MJ/kg	Proximate Analysis (%)			Ultimate Analysis (%)					Ref.
	HHV	VM	FC	ASH	C	H	N	O	S	
Acacia mangium (Hardwood)	19.50	83.94	14.94	1.12	48.93	6.06	0.43	43.44	0.02	[19]
Alabama oak wood waste	19.23	74.70	21.90	3.30	49.50	5.70	0.20	41.30	0.00	[7]
Alfalfa stems	18.67	78.92	15.81	5.27	47.17	5.99	2.68	38.19	0.20	[9]
Almond	20.01	76.83	21.54	1.63	51.30	5.29	0.66	40.90	0.01	[7]
Almond hulls	18.89	73.80	20.07	6.13	47.53	5.97	1.13	39.16	0.06	[9]
Almond shells	19.49	76.00	20.71	3.29	49.30	5.97	0.76	40.63	0.04	[9]
Akhrot shell	20.01	80.55	18.65	0.71	50.22	5.70	0.00	43.37	0.00	[2]
Ash wood	18.10	82.10	16.20	1.70	49.20	5.60	0.20	43.30	0.00	[20]
Bagasse	17.33	73.78	14.95	11.27	44.80	5.35	0.38	38.55	0.01	[2]
Bamboo	17.55	75.83	20.55	3.62	43.84	6.05	0.07	46.53	0.00	[48]
Bamboo (*Bambusa vulgaris*) bottom	18.32	79.86	18.19	1.95	46.01	6.24	0.18	45.63	0.00	[49]
Bamboo (*Bambusa vulgaris*) middle	18.47	81.14	17.43	1.43	46.37	6.33	0.18	45.70	0.00	[49]
Bamboo (*Bambusa vulgaris*) mixture	18.21	80.13	17.75	2.12	45.79	6.24	0.24	45.60	0.00	[49]
Bamboo (*Bambusa vulgaris*) top	18.37	79.08	18.52	2.40	45.35	6.19	0.26	45.79	0.00	[49]
Bamboo leaf	17.76	71.67	8.31	20.02	39.81	5.68	3.26	31.32	0.00	[50]
Bamboo wood	20.55	86.80	11.24	1.95	48.76	6.32	0.20	42.77	0.00	[2]
Banana leaf	19.18	80.35	9.34	10.31	44.54	5.74	1.90	37.52	0.00	[50]
Banyan	18.46	80.21	18.64	1.15	46.20	6.08	0.08	46.53	0.00	[48]
Biomass mix	18.40	69.36	18.14	12.49	49.59	5.79	2.43	28.87	0.74	[9]
Birch	19.94	89.46	10.26	0.28	48.49	6.35	0.11	44.60	0.02	[21]
Birch	19.50	89.74	9.63	0.64	48.24	6.15	0.16	44.80	0.02	[22]
Birch (Norwegian)	20.36	89.46	10.26	0.28	48.94	6.35	0.11	44.60	0.02	[51]
Block wood	18.26	83.32	14.59	2.09	46.90	6.07	0.95	43.99	0.00	[7]
B-wood	20.05	76.53	21.62	1.85	50.26	6.91	1.03	39.66	0.00	[9]
Cabernet sauvignon	19.03	78.63	19.20	2.17	46.59	5.85	0.83	44.90	0.04	[7]
Camphorwood	18.86	85.51	13.42	1.07	47.66	6.02	0.69	44.55	0.00	[23]
Canyon live oak	18.98	88.20	11.30	0.50	47.84	5.80	0.07	45.76	0.01	[7]
Cassava rhizome	17.22	76.00	19.00	5.00	49.20	6.70	1.00	38.00	0.11	[12]
Caster bean cake	19.12	69.80	21.41	8.79	40.32	5.41	7.22	38.25	0.00	[23]
Casuarina equisetifolia leaf	18.48	79.50	16.46	3.93	46.12	6.90	1.18	41.84	0.00	[9]
Casuarina wood	18.77	78.58	19.58	1.83	48.50	6.24	0.31	43.12	0.00	[2]
Cedar wood	19.57	81.86	17.25	0.89	48.51	5.86	0.64	44.09	0.00	[23]
Chaparral wood	18.61	75.19	18.68	6.13	46.90	5.08	0.54	41.17	0.03	[2]
Coconut coir	20.05	66.58	29.70	3.72	50.29	5.05	0.45	40.63	0.16	[7]
Coconut coir	19.08	79.23	17.85	2.92	46.33	3.74	1.46	45.56	0.00	[50]
Coconut coir pitch	18.07	66.02	28.82	5.16	43.36	4.98	1.63	44.87	0.00	[2]
Coconut leaf	20.83	80.75	12.92	6.33	47.89	6.19	1.66	37.93	0.00	[50]
Coconut shell	20.50	77.19	22.10	0.71	50.22	5.70	0.00	43.37	0.00	[2]
Coffee husk	19.80	78.50	19.10	2.40	47.50	6.40	0.00	43.70	0.00	[9]
Corn stover	17.93	66.58	26.65	6.73	45.48	5.52	0.69	41.52	0.04	[9]
Corn straw	17.68	73.15	19.19	7.65	44.73	5.87	0.60	41.04	0.07	[9]
Corncob	19.06	80.50	17.50	2.00	49.70	5.90	0.38	42.00	0.03	[12]
Corncob	18.51	80.10	17.80	2.10	46.60	5.97	0.49	44.84	0.00	[39]
Corncob	18.77	80.10	18.54	1.36	46.58	5.87	0.47	45.46	0.01	[7]
Corncob	17.00	86.50	12.50	1.00	49.00	5.40	0.04	44.60	0.00	[7]
Corncob	18.76	88.25	9.51	2.23	43.77	4.67	1.17	48.16	0.00	[50]

Cotton gin waste	17.48	83.41	14.97	1.61	42.66	6.05	0.18	49.50	0.00	[7]
Cotton residue	16.90	72.80	20.59	6.61	47.30	5.96	1.79	38.42	0.19	[9]
Cotton stalk	16.90	76.80	16.80	6.45	42.10	5.64	0.22	45.28	0.28	[24]
Cotton stalk	15.83	65.40	17.30	17.30	39.47	5.07	1.20	37.14	0.02	[7]
Cotton stalk	17.40	76.10	18.80	5.10	47.07	4.58	1.15	42.10	0.00	[9]
Cotton stalk (dried)	16.53	75.38	18.09	6.53	48.43	6.93	0.93	37.18	0.00	[25]
Cotton stalk briquette	18.64	79.13	17.50	3.37	49.93	5.73	0.27	39.16	1.54	[26]
Cotton stalk particle	19.32	82.34	15.73	1.93	50.72	5.32	0.46	41.50	0.07	[26]
Cotton gin trash	16.42	67.30	15.10	17.60	39.59	5.26	2.09	35.38	0.00	[7]
Dal lake weed	8.89	47.70	3.60	48.70	19.12	2.00	4.22	25.96	0.00	[7]
Douglas fir pellet	20.38	79.93	19.85	0.22	47.90	6.55	0.08	45.57	0.00	[52]
Douglass fir	22.10	73.00	25.80	1.20	56.20	5.90	0.00	36.70	0.00	[7]
Douglass fir wood	20.38	87.30	12.60	0.10	50.64	6.18	0.06	43.00	0.02	[2]
Dried grains-solubles	21.75	82.50	13.64	3.89	50.24	6.89	4.79	33.42	0.77	[9]
Esparto plant	19.10	80.50	16.80	2.20	46.94	6.44	0.86	43.56	0.00	[9]
Eucalyptus saligna (hardwood)	19.60	82.55	16.66	0.79	49.79	6.02	0.37	42.95	0.08	[19]
Eucalyptus	18.40	81.10	18.90	0.01	50.79	5.10	0.00	44.10	0.00	[28]
Eucalyptus	20.17	79.11	19.29	1.60	48.51	6.40	0.00	43.59	0.00	[27]
Eucalyptus globulus wood	19.23	81.60	17.30	1.10	48.18	5.92	0.39	44.18	0.01	[2]
Eucalyptus grandis	19.35	82.55	16.93	0.52	48.33	5.89	0.15	45.13	0.01	[7]
Eucalyptus saw dust	18.50	83.60	16.20	0.20	49.37	6.40	2.02	42.01	0.00	[7]
Eucalyptus wood	18.64	75.35	21.30	3.35	46.04	5.82	0.30	44.49	0.00	[7]
Grape pomace	20.04	72.60	21.90	5.40	46.45	5.96	2.17	40.03	0.00	[30]
Groundnut shell	19.85	72.70	21.60	5.70	48.59	5.64	0.58	39.49	0.00	[2]
Hardwood (mixture of oak and birch)	18.17	82.62	16.78	0.70	46.47	5.86	0.10	46.87	0.00	[27]
Hardwood chips	19.48	84.74	14.83	0.65	55.04	5.36	0.01	38.95	0.00	[53]
Hazelnut shell	19.90	68.90	30.00	1.10	50.34	5.84	0.40	42.33	0.00	[9]
Ipil ipil	20.22	79.90	17.70	2.40	48.30	6.80	0.00	42.50	0.00	[9]
Jackfruit leaf	17.03	70.05	13.67	16.28	42.33	4.70	3.12	33.57	0.00	[50]
Jawar straw	17.95	75.97	15.15	8.88	42.10	5.60	0.04	43.38	0.00	[2]
Jujuba wood	19.77	83.63	14.14	2.32	47.63	6.12	0.15	43.78	0.00	[2]
Juniper	20.18	82.85	15.19	2.03	52.36	6.03	0.30	39.30	0.01	[31]
Lauan wood	20.41	81.12	18.61	0.27	48.64	6.75	0.10	44.24	0.00	[54]
Lawn grass	17.66	74.94	12.39	12.67	37.79	5.46	2.34	41.74	0.00	[50]
Leucaena	20.30	86.10	13.10	0.80	49.70	7.34	0.69	41.47	0.00	[33]
Leucaena	20.30	87.30	12.20	0.50	49.85	7.36	0.63	41.59	0.00	[32]
Loblolly pine	21.77	65.70	33.90	0.40	56.30	5.60	0.00	37.70	0.00	[7]
Loblolly pine chips	20.00	84.60	14.80	0.60	50.50	6.26	0.09	42.60	0.00	[34]
Macadamia shell	21.01	75.92	23.68	0.40	54.41	4.99	0.36	39.69	0.01	[7]
Mango leaf	18.83	73.87	14.75	11.38	43.89	4.57	1.59	38.57	0.00	[50]
Mango wood	19.17	85.64	11.36	2.98	46.24	6.08	0.28	44.42	0.00	[2]
Marabu	20.72	81.30	17.20	1.50	48.60	6.30	0.00	43.60	0.00	[9]
Mast tree leaf	22.37	84.00	10.74	5.26	50.60	5.93	2.69	35.52	0.00	[50]
Mesquite	19.76	78.22	19.76	1.98	51.60	5.89	0.73	39.76	0.04	[31]
Mulberry stick	18.36	75.10	22.80	2.10	44.23	6.61	0.51	46.25	0.00	[7]
Millet grain waste	15.21	77.10	14.50	8.40	40.56	5.26	0.40	45.50	0.00	[2]
Millet straw	18.05	78.28	16.45	5.27	43.71	5.85	0.01	45.16	0.00	[2]
Miscanthus	17.90	82.20	15.10	2.70	48.40	5.80	0.20	42.90	0.00	[20]
Miscanthus	17.47	87.50	11.70	0.80	46.70	6.00	0.20	45.67	0.00	[35]
Miscanthus	11.81	54.40	5.70	39.90	30.11	3.97	0.84	25.18	0.00	[39]
Miscanthus giganteus	18.36	91.16	4.36	4.47	45.50	5.91	0.38	43.73	0.00	[37]
Narrow-leaf cattail	16.27	79.19	10.39	10.42	39.66	5.18	0.93	43.81	0.00	[50]
Neem wood	20.26	85.86	12.19	1.93	48.26	6.27	0.08	43.46	0.00	[2]
Oak wood (large branch)	19.17	81.75	16.18	2.07	47.56	6.67	2.34	41.36	0.00	[9]
Oak wood (medium branch)	19.24	80.82	16.18	3.00	47.16	6.32	2.50	41.01	0.00	[9]
Oak wood (small branch)	19.20	77.45	18.50	4.05	48.76	6.35	2.70	40.38	0.00	[9]
Oil palm fiber	17.10	72.50	20.50	7.00	48.27	4.46	2.23	37.76	0.28	[28]
Oil palm frond	18.21	79.10	16.90	4.00	44.80	5.50	0.31	45.30	0.12	[12]
Oil palm fruit bunch	16.96	79.00	16.46	4.53	45.90	5.80	1.20	42.60	0.00	[9]
Olive cake	21.60	62.10	34.60	2.80	53.70	6.70	0.60	36.20	0.00	[9]
Olive kernel	22.40	65.01	33.37	1.70	54.60	6.80	0.80	36.10	0.00	[9]
Olive kernel	19.90	73.62	24.25	2.13	52.44	6.17	1.32	37.85	0.09	[9]

Olive kernel shell	21.40	60.50	36.10	3.30	53.20	6.70	0.50	36.30	0.00	[9]
Olive pits	21.59	82.00	16.28	1.72	52.80	6.69	0.45	38.25	0.05	[9]
Olive stone (*Olive europaea*)	20.50	82.41	17.22	0.37	51.62	5.97	0.25	41.78	0.01	[19]
Olive stones	17.54	63.80	21.74	14.46	36.95	4.79	1.63	42.17	0.00	[38]
Paddy straw	14.52	72.70	11.80	15.50	35.97	5.28	0.17	43.08	0.00	[7]
Palm kernel shells	20.42	75.20	18.30	6.50	50.86	5.52	0.37	36.75	0.00	[39]
Palm kernel shells	17.93	75.00	20.58	4.40	51.83	6.28	0.44	37.03	0.00	[40]
Palm kernel shells	20.71	77.28	17.59	5.14	48.34	6.20	2.62	37.44	0.26	[9]
Peach pit	19.42	79.10	19.80	1.10	48.60	6.27	0.47	43.04	0.02	[7]
Peanut shell	18.60	84.90	13.40	1.70	46.59	6.00	2.06	43.65	0.00	[9]
Pennisetum	16.98	93.34	0.67	5.99	43.74	5.98	0.24	44.06	0.00	[55]
Pepper plant	15.39	64.71	20.86	14.44	36.11	4.26	2.72	41.86	0.49	[9]
Pine	19.20	86.70	12.90	0.40	51.30	6.00	0.10	42.30	0.00	[20]
Pine	18.44	92.23	7.71	0.05	46.73	6.46	0.41	46.35	0.00	[41]
Pine	18.44	92.23	7.71	0.05	46.73	6.46	0.41	46.35	0.00	[41]
Pine chips	19.79	72.40	21.65	5.95	49.66	5.67	0.51	38.07	0.08	[9]
Pine chips	18.46	85.98	13.76	0.27	47.21	6.64	0.17	45.76	0.00	[56]
Pine shells	18.82	68.41	30.08	1.51	47.08	5.52	0.30	45.60	0.00	[38]
Pinus illiottis (softwood)	20.00	82.10	17.20	0.70	51.10	6.14	0.41	41.56	0.09	[19]
Pistachio shell	19.26	82.03	16.84	1.13	48.79	5.91	0.56	43.41	0.01	[7]
Pistachio shell	18.22	81.64	16.95	1.41	50.20	6.32	0.69	41.15	0.22	[9]
Plywood	18.96	82.14	15.77	2.09	48.13	5.87	1.45	42.46	0.00	[2]
Poplar (hybrid)	19.02	84.81	12.49	2.70	50.18	6.06	0.60	40.43	0.02	[9]
Rape stalk	18.98	77.27	19.53	3.20	47.53	6.20	0.37	42.46	0.00	[57]
Rape straw	18.34	76.54	18.81	4.65	46.17	6.12	0.46	42.47	0.10	[9]
Red wood	20.72	79.72	19.92	0.36	50.64	5.98	0.05	42.88	0.03	[7]
Refused derived fuel	19.50	72.00	17.60	10.40	44.72	6.21	0.69	38.36	0.00	[2]
Residue (*Eucalyptus saligna*)	19.50	80.98	17.76	1.26	49.66	6.09	0.54	42.39	0.06	[19]
Residue (forest)	19.50	79.80	20.00	0.20	53.16	6.25	0.30	40.00	0.09	[9]
Residue (logging chips)	18.79	82.17	16.07	1.77	47.29	6.20	0.42	44.19	0.00	[56]
Residue 1 (forest)	18.80	83.00	15.10	1.90	50.82	5.69	0.00	41.50	0.00	[58]
Residue 2 (forest)	19.00	80.30	15.00	4.70	48.70	5.53	0.00	41.07	0.00	[58]
Residue 3 (forest)	18.70	82.40	15.90	1.70	50.23	5.70	0.00	42.37	0.00	[58]
Residue 4 (forest)	19.40	82.90	14.50	2.60	49.87	5.75	0.00	41.78	0.00	[58]
Residue 5 (forest)	18.60	79.30	17.80	2.90	50.49	5.73	0.00	40.88	0.00	[58]
Residues (Norwegian forest)	19.52	74.74	22.94	2.34	47.58	6.10	0.48	43.62	0.07	[42]
Rice husk	15.16	64.89	19.83	15.23	42.13	5.40	0.55	36.47	0.17	[43]
Rice husk	16.58	70.60	12.87	16.53	39.37	5.13	0.32	38.59	0.06	[44]
Rice husk	14.69	61.81	16.95	21.24	38.50	5.20	0.45	34.61	0.00	[2]
Rice husk	16.47	61.20	16.30	22.50	38.20	5.60	0.00	33.70	0.00	[9]
Rice husk	17.40	80.45	8.60	10.95	41.20	6.13	0.22	41.11	0.00	[59]
Rice husk bran	15.29	61.83	19.53	18.64	38.92	5.12	0.55	36.77	0.00	[2]
Rice husk briquette	16.01	68.05	16.03	15.92	45.02	4.05	0.61	34.30	0.09	[26]
Rice husk particle	16.36	82.77	5.84	11.39	45.93	5.02	0.38	37.03	0.24	[26]
Rice husk patni-23	15.67	69.30	14.90	15.80	38.92	5.10	2.17	37.89	0.12	[2]
Rice husks	16.67	68.00	15.40	16.60	41.20	5.25	0.42	36.53	0.00	[39]
Rice straw	17.20	71.80	17.00	11.20	45.30	5.90	0.88	36.60	0.15	[12]
Rice straw	19.00	79.60	15.90	4.09	43.16	5.78	0.22	46.42	0.29	[24]
Rice straw	14.85	65.70	13.91	20.38	35.68	4.62	0.28	39.14	0.00	[2]
Rice straw	15.09	65.47	15.86	18.67	38.24	5.20	0.87	37.26	0.18	[9]
Rice straw	15.07	63.61	6.88	29.51	34.81	4.41	1.21	30.07	0.00	[50]
Rice straw	17.10	71.70	18.58	9.72	43.68	5.70	0.97	39.72	0.21	[60]
Rice straw	16.16	88.78	1.43	9.82	41.70	5.60	1.19	41.69	0.00	[55]
Rice straw	18.04	72.54	18.40	9.05	41.10	5.35	1.09	43.17	0.00	[57]
Sal seed husk	20.60	62.54	28.06	9.40	48.12	6.55	0.00	35.93	0.00	[7]
Senna leaves	18.13	57.20	25.50	17.30	36.20	4.72	4.29	37.49	0.00	[7]
Shea meal	19.80	66.30	28.70	5.00	48.56	5.86	2.88	37.70	0.00	[9]
Softwood (mixture of spruce, pine and larch)	18.38	82.92	16.98	0.10	46.65	5.89	0.00	47.35	0.00	[27]
Soplillo	22.58	77.80	20.70	1.50	48.80	6.50	0.00	43.20	0.00	[9]
Spire-mint	15.53	70.10	11.80	18.10	37.23	5.34	5.95	33.38	0.00	[7]
Spruce	20.42	86.50	13.27	0.23	50.31	6.24	0.07	43.38	0.00	[45]
Spruce	20.42	86.50	13.27	0.23	50.31	6.24	0.07	43.38	0.02	[21]
Spruce	20.70	85.18	14.14	0.68	50.96	6.18	0.12	42.06	0.02	[22]

Spruce (Norwegian)	19.89	86.50	13.27	0.23	50.31	6.24	0.07	43.38	0.02	[51]
Spruce (Norwegian)	19.05	81.24	18.38	0.38	47.20	6.21	0.02	46.18	0.03	[42]
Spruce wood	20.29	85.00	14.60	0.40	50.40	5.90	0.10	43.20	0.00	[46]
Subabul fresh wood	19.70	83.60	15.20	1.12	46.24	5.80	0.25	46.59	0.00	[2]
Subabul wood	19.78	80.05	18.30	1.20	48.15	5.87	0.03	44.75	0.00	[7]
Sudan grass	17.39	72.75	18.60	8.65	44.58	5.35	1.21	40.18	0.01	[7]
Sugar cane	17.94	73.80	17.90	8.30	47.40	5.90	0.55	37.70	0.15	[12]
Sugar cane leaves	17.41	77.40	14.90	7.70	39.75	5.55	0.17	46.82	0.00	[7]
Sugar cane straw	17.19	76.20	14.60	9.20	43.50	6.10	0.00	41.10	0.00	[9]
Sugarcane bagasse	18.37	83.66	13.15	3.20	45.48	5.96	0.15	45.21	0.00	[2]
Sugarcane bagasse	17.70	81.50	13.30	5.20	43.79	5.96	1.69	43.36	0.00	[9]
Sugarcane bagasse	17.32	82.60	14.70	2.70	47.20	7.00	0.00	43.10	0.00	[9]
Sugarcane residue	17.80	93.60	3.65	2.50	49.19	6.94	0.43	40.45	0.00	[59]
Sunflower seed shell	17.60	84.70	11.70	3.60	49.84	5.98	0.96	39.62	0.00	[9]
Sweet sorghum bagasse	18.68	79.16	16.20	4.64	41.48	5.34	0.27	48.15	0.12	[53]
Switch grass	20.60	80.63	15.87	3.50	45.71	6.38	0.60	42.43	1.38	[47]
Switch grass	18.06	76.69	14.34	8.97	46.68	5.82	0.77	37.38	0.19	[9]
Switch grass (densified raw)	19.14	80.23	16.15	3.62	45.40	5.72	0.28	43.80	1.20	[47]
Tan oak	18.93	90.60	9.20	0.20	48.67	6.03	0.06	44.99	0.04	[7]
Tea bush	19.84	76.50	21.80	1.70	47.67	6.13	1.33	43.16	0.00	[7]
Tea waste	17.10	85.00	13.60	1.40	48.60	5.50	0.50	44.00	0.00	[7]
Vine prunings	19.25	80.80	16.60	2.60	49.48	5.65	0.88	41.40	0.00	[39]
Walnut	19.97	78.50	20.80	0.70	48.20	6.25	1.61	43.24	0.00	[7]
Water hyacinth	16.65	76.85	12.17	10.98	37.42	3.30	2.04	46.27	0.00	[50]
Western hemlock wood	20.05	82.93	14.87	2.20	50.40	5.80	0.10	41.80	0.10	[2]
Wet grains	21.95	83.18	14.28	2.58	52.53	6.60	5.35	32.28	0.66	[9]
Wheat straw	16.80	76.30	15.70	8.00	45.30	5.60	0.90	40.20	0.00	[20]
Wheat straw	19.00	71.24	12.62	16.14	33.04	4.36	0.42	46.04	0.00	[38]
Wheat straw	17.00	63.00	23.50	13.50	45.50	5.10	1.80	34.10	0.00	[7]
Wheat straw	17.99	82.12	10.98	6.90	42.95	5.35	0.00	44.99	0.00	[2]
White fir	19.95	83.17	16.58	0.25	49.00	5.98	0.05	44.75	0.01	[7]
Willow	19.30	83.98	15.12	0.50	49.25	6.07	0.20	44.18	0.00	[27]
Willow	18.45	83.18	16.13	0.69	45.80	6.24	0.09	47.20	0.00	[48]
Willow wood	19.59	82.22	16.07	1.71	49.90	5.90	0.61	41.80	0.07	[9]
Wood chips	19.92	76.40	23.50	0.10	48.10	5.99	0.08	45.74	0.00	[7]

© 2017 by the authors; licensee MDPI, Basel, Switzerland. This article is an open access article distributed under the terms and conditions of the Creative Commons Attribution (CC BY) license (http://creativecommons.org/licenses/by/4.0/).
